# Angry Women Are More Trusting: The Differential Effects of Perceived Social Distance on Trust Behavior

**DOI:** 10.3389/fpsyg.2021.591312

**Published:** 2021-07-21

**Authors:** Keshun Zhang, Thomas Goetz, Fadong Chen, Anna Sverdlik

**Affiliations:** ^1^Department of Psychology, Qingdao Psychological and Mental Health Research Institute, Qingdao University, Qingdao, China; ^2^Graduate School of Decision Sciences, University of Konstanz, Konstanz, Germany; ^3^Department of Developmental and Educational Psychology, Faculty of Psychology, University of Vienna, Vienna, Austria; ^4^School of Management and Neuromanagement Lab, Zhejiang University, Hangzhou, China; ^5^Department of Educational and Counselling Psychology, McGill University, Montreal, QC, Canada

**Keywords:** trust, anger, gender, social distance, motivation

## Abstract

Accumulating evidence suggests that anger can have a strong impact on discrete trust behaviors. However, the mechanisms underlying how anger influences trust are still unclear. Based on the appraisal tendency framework, we hypothesized that perceived social distance would positively mediate the effect of anger on trust, and that gender would moderate this mediation. In Study 1, a 2 (Anger vs. Control) × 2 (Men vs. Women) factorial design was used to investigate this hypothesis. Results supported our predictions that anger drove women, but not men, to perceive smaller social distance, and thus sent more money to their counterparts in a trust game as compared to controls. In Study 2, social distance was manipulated, and a 2 (Low social distance vs. Control) × 2 (Men vs. Women) factorial design was used to critically test the causal role of the mediator, namely to examine the effect of perceived social distance on trust. Results showed that women, but not men, sent more money to their counterparts in the low social distance condition than in the control condition. Results of both studies indicate that the high certainty, higher individual control, and approach motivation associated with anger could trigger optimistic risk assessment, and thus more trust toward others in women, via perceiving smaller social distance to others.

## Introduction

Trust permeates not only interpersonal interactions, but also can be a cornerstone of economic transactions (Kramer, 1998; Greenspan, 1999; Zak and Knack, 2001; Algan and Cahuc, 2013). Traditionally trust has been viewed as a deliberate act based on thorough cognitive calculations (Williamson, 1993; Kramer, 1998). However, recent behavioral studies suggest that trust-related behaviors involve a variety of emotions, thereby going beyond mere cognitive calculations (Fehr et al., 2005; Bohnet et al., 2008; Engelmann and Fehr, 2013; Lerner et al., 2015).

In the present study, we develop a theoretical approach based on the Appraisal Tendency Framework (ATF; Lerner and Keltner, 2000, 2001; Han et al., 2007), which is a general theoretical model predicting how specific emotions impact economic judgments and choices. To date, an increasing number of empirical studies have investigated the impact of emotions on trust behavior (e.g., Dunn and Schweitzer, 2005; Myers and Tingley, 2016). As anger is one of the most frequently experienced emotions in our daily life (Averill, 2012), it merits special attention in the process of trust. Anger affects basic cognitive (e.g., perceptions of control and certainty, Lerner and Tiedens, 2006) and social processes (e.g., Fischer and Roseman, 2007; Fischer and Manstead, 2008; Zhang et al., 2020) which, in turn, can influence the trust decisions people make. We will examine the way in which anger, as a specific emotion, influences trust in the present study. Further, increasing evidence suggests that the perceived social distance (i.e. subjectively perceived distance to other people) to the trustee may positively mediate the influence of anger on trust (Forgas, 1995; Dunn and Schweitzer, 2005; Buchan et al., 2008). For this reason, we will test the mediating role of social distance in the present work. In addition, research has shown that women more frequently use social cues (e.g., emotions) to determine their trust levels than men (Croson and Gneezy, 2009). Therefore, we will critically test the role of gender and social distance as factors shaping the effect of anger on trust. Thus, we propose a moderated mediation model in which gender plays a moderating role and perceived social distance plays a positive mediating role in the relationship between anger and trust, and consequently aim to contribute to the understanding of the mechanisms by which anger impact trust behavior. In the following sections, we will briefly review the theoretical framework and empirical evidence outlining how anger influence trust.

### The Relationship Between Anger and Trust

The ATF assumes that “each emotion activates a cognitive predisposition to appraise future events in line with the central appraisal dimensions that triggered the emotion” (Lerner and Keltner, 2000, p. 477). Specific emotions give rise to specific cognitive and motivational processes, which guide subsequent behavior and cognitions (Lerner et al., 2007). To yield a strong influence, the emotion's central appraisal content must be thematically linked to the decision-making topic (Lerner and Keltner, 2001). Previous studies have identified three central dimensions of emotions that can be used to distinguish the effects of discrete emotions on judgments and choices. These dimensions are *control, certainty*, and *approach vs. avoidance motivation* (e.g., Lerner and Keltner, 2001; Dunn and Schweitzer, 2005; Carver and Harmon-Jones, 2009). Specifically, *control* is the degree to which events seem to be caused by situational agency (low control) vs. individual agency (high control), *certainty* is the degree to which future events seem unpredictable (low certainty) vs. predictable (high certainty), and *associated motivation* is the urge to avoid or approach a stimulus (Angus et al., 2015; Zhang et al., 2020). For example, anger arises as a result of perceiving obstacles which inhibit progress toward a desired target (Berkowitz, 1993), and is associated with the appraisal that another person is responsible for the negative occurrence. At the same time, people believe that they by themselves can still influence the situation (e.g., Scherer, 2001; Lerner and Tiedens, 2006). Therefore, anger, although of negative valence, is associated with high certainty and high individual control (Smith and Ellsworth, 1985). Thus anger was found to drive people to make optimistic risk estimates in a lottery-based risk game (Lerner and Keltner, 2001). In contrast, fear involves low certainty and a low sense of control, which leads fearful people to see greater risks. Furthermore, anger is associated with approach motivation which facilitates the pursuit of rewarding stimuli; however, fear is associated with avoidance motivation which should lead people to avoid risks (Carver and Harmon-Jones, 2009; Angus et al., 2015).

Trust is defined as “a psychological state comprising the intention to accept vulnerability based upon positive expectations of the intentions or the behavior of another” (Rousseau et al., 1998, p. 395) reflecting a typical person-based risk (i.e., where risk results from the uncertain behavior of another person). In contrast to this definition, existing research on emotions and risk has mainly involved lottery-based risk (with known probabilities and profit outcomes). Even though the research on the effects of emotions on person-based risk is limited (Croson and Gneezy, 2009; Kugler et al., 2012), previous evidence indicated that people behaved differently in response to these two sources of risk (Kugler et al., 2012; Schlösser et al., 2015). Therefore, in order to test whether there are distinct effects of specific emotions on person-based risk as compared to lottery-based risk, we will examine the impact of anger on trust. We expect the above three appraisal dimensions of emotions (*control, certainty*, and *approach vs. avoidance motivation*) to be particularly influential, due to their close association with cognitive evaluations for determining trust-related decisions. Specifically, the associated high certainty, high individual control, and approach motivation of anger could drive people to make optimistic risk estimates and to be less risk averse in the context of trust decisions (Lerner and Keltner, 2001; Angus et al., 2015; Beisswingert et al., 2015). Hence, we predict that angry people will demonstrate more reward-seeking tendencies, and thus trust others more.

### Moderated Mediation Model on the Impact of Anger on Trust: The Role of Social Distance and Gender

Furthermore, we propose that the perceived social distance to the trustee will mediate the influence of anger on trust (Forgas, 1995; Dunn and Schweitzer, 2005). Social distance is defined as the perceived distance, or perceived dimension of closeness between interacting individuals or groups (Dufwenberg and Muren, 2006; Fiedler et al., 2011). Previous studies have shown that emotions have a variety of social functions, for example, by increasing or decreasing the perceived social distance between the self and others (e.g., Fischer and Roseman, 2007). Anger increased perceived social distance between the self and the target of the anger (Fischer and Manstead, 2008), but could decrease social distance between the self and others who are not the source of anger (e.g., Archer and Coyne, 2005). 70 million tweets indicated that anger could trigger people to perceive less social distance toward their network partners on the online social media who were not the source of their anger than joy and sadness, which might be due to the relationship between anger and approach motivation (Fan et al., 2014). Therefore, we assume that the associated motivation of specific emotions can shape the perceived social distance to others. Specifically, anger associated with approach motivation, might decrease one's perceived social distance to those not responsible for the anger. Perceived social distance has recently been acknowledged to have an important influence on trust behavior (Fiedler et al., 2011; Binzel and Fehr, 2013). A decrease in perceived social distance should lead to more trust behavior (Fiedler et al., 2011). For example, American participants send more money when matched with an ingroup member than with an outgroup member in a trust game (Buchan et al., 2008). By considering the effect of anger on social distance and the effect of social distance on trust, we therefore propose a positive mediating role of social distance in the relationship between anger and trust.

In addition to the mediating role of social distance, we propose that gender moderates the effects of anger on trust (Croson and Gneezy, 2009; Ferrer et al., 2016; Rand et al., 2016). Social role theory emphasizes that the female role promotes communal behavior, and the male gender role promotes agentic behavior (von Neumann and behavior, 1944). Therefore, women were more sensitive to social cues in determining proper behavior than are men (Gilligan, 1982). Various findings show gender differences in trust behavior. For example, women's trust varied to a greater extent than men's based on the social cues perceived during interactions (e.g., von Neumann and Morgenstern, 1944; Buchan et al., 2008). Evidences suggests that gender differences in trust behavior might be related to perceived social distance, as women were more likely to be dependent on their perceived social distance to frame their choices in the investment game than men (e.g., Cox and Deck, 2006; Croson and Gneezy, 2009; Zhang et al., 2020). Women are therefore more sensitive to perceived social distance, which in turn should affect their trust, whereas this factor should have less impact in men's trust. In the present study, we propose a moderated mediation model in which perceived social distance would positively mediate the effect of anger on trust, and gender would moderate this mediation. More specifically, we predict anger will decrease women's, but not men's, perception of social distance from their game partners, as compared to women in an emotion neutral condition, which should then increase trust.

### The Present Research

**Figure 1:**
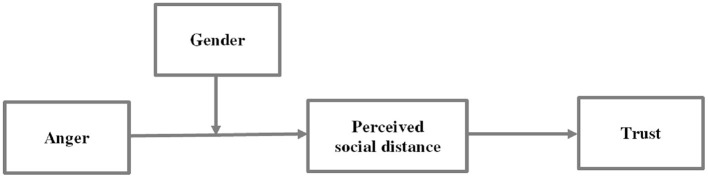
Conceptual moderated mediation model.

The objectives of the present study are twofold: First, we will investigate the impact of anger, as caused by another person, on trust. We will examine the assumed moderated mediation model (Figure 1), that the effect of anger on trust is mediated by perceived social distance, and this mediation is moderated by gender. More precisely, based on the associated high certainty, higher individual control, approach motivation of anger, and the potential effect of social distance and gender (e.g., Lerner and Keltner, 2001; Buchan et al., 2006; Croson and Gneezy, 2009), we hypothesize that women, but not men, are driven by anger to trust their counterparts more than controls (Hypothesis 1). Second, we will critically test the causal role of the mediator, namely to examine the effect of perceived social distance on trust, utilizing an experimental approach to manipulate perceived social distance (i.e., low social distance vs. control). We predict that women, but not men, send more money to their counterparts in the low social distance condition than in the control condition (Hypothesis 2).

The first aim is to adopt an appropriate experimental method for inducing anger as caused by another person in the pilot study. Next, the hypotheses concerning the effects of anger, social distance and gender on trust behavior are investigated in the Study 1 and Study 2.

## Pilot Study

The objective of this pilot experimental study was to adopt a directed writing task to manipulate anger (Strack et al., 1985), known as the “Autobiographical Emotional Memory Task” (AEMT, Mills and D'Mello, 2014), which was proved to be efficient in inducing anger with cross-cultural generalizability (Zhang et al., 2020). This pilot study was planned as a manipulation check in order to ensure that the anger was successfully aroused in the experimental condition as compared to a control condition.

### Method

#### Participants and Data Collection

Thirty-Two German university students (78.1% female) with an average age of *M* = 23.41 years (*SD* = 2.75, range: 20–29) voluntarily participated in this study. The participants were recruited using the online recruiting system ORSEE (Greiner, 2015) and they were compensated with 7.50 €. The pilot experiment lasted for half an hour. The assignment to the treatment conditions was random with *n* = 16 participants in the *anger* and *n* = 16 in the *control* condition.

#### Experimental Design and Procedure

The experiment consisted of a one-factor repeated-measure design. Participants completed the emotion rating survey before and after writing the AEMT (pre- and post-emotion ratings, respectively). After reading paper instructions of the experiment, participants were asked to answer two control computer-based questions about the experiment to ensure appropriate understanding of the task, followed by the pre-writing emotion rating assessment. Next, the AEMT was used to manipulate anger. The AEMT involved recalling and writing in detail about intense emotional experiences. This study adopted the AEMT to elicit anger, and further specified the sources of anger as another person. Therefore, participants in the anger condition were asked to describe an anger-filled event with the following instruction: “*Please describe in detail the one situation caused by* ***another person*** *(not yourself) that has made you the most* ***angry*** *you have ever been in your life, and vividly describe how the event occurred. Please describe it such that a person reading the description would become [**angry**] just from hearing about the situation.”* While in the control condition, participants were asked to “*Describe in detail the* ***mundane events*** *of the previous day”* (Bodenhausen et al., 2000). Participants typed their responses on the computer and the content of their responses was stored for offline analysis. Participants were advised to finish writing in 6 mins and that they could continue to write for an extra 2 mins if they didn't finish in the allotted time. Participants completed the post-writing emotion assessment after finishing the AEMT. Social-demographic variables (e.g., sex, age, subject of study) were assessed at the end of the experiment.

#### Variables and Study Measures

##### Emotion

Using the subscale of the Differential Emotion Scale (Izard et al., 1974; German version: Merten and Krause, 1993), the Academic Emotions Questionnaire (Pekrun et al., 2002) and the PANAS-X (German version, Röcke and Grühn, 2003; English version, Watson and Clark, 1999), anger (the target emotion) and nine other emotions (anxiety, fear, sadness, shame, hopelessness, boredom, enjoyment, pride, and hope) were assessed, in order to be able to judge whether our manipulation indeed had an effect on experienced anger. Each emotion consisted of three adjective items (e.g., anger: “enraged”, “angry”, “mad”). Participants' self-reported emotions were assessed on a five-point intensity rating scale ranging from 0 *not at all* to 4 *very strong*. The internal consistencies of these ten emotions at both time points were: anger (0.85/0.96), anxiety (0.84/0.87), fear (0.79/0.75), sadness (0.79/0.90), shame (0.70/0.82), hopelessness (0.71/0.82), boredom (0.45/0.61), enjoyment (0.87/0.85), pride (0.74/0.85), and hope (0.82/0.87).

### Results and Discussion

#### Pre-writing Levels of Emotions

In the baseline, there were no significant group differences in anger between the anger and control conditions, *t* (30) = −1.67, *p* = 0.105 (Anger: *M* = 0.15, *SD* = 0.27; Control: *M* = 0.46, *SD* = 0.70), and there were no significant group differences in the other nine emotions (*p* > 0.05 in all the *t*-tests).

#### Post-writing Levels of Emotions

Following the experimental manipulation, participants in the anger condition showed significantly higher levels of anger than the participants in the control condition, *t* (30) = 3.06, *p* = 0.005 (Anger: *M* = 1.90, *SD* = 1.54; Control: *M* = 0.58, *SD* = 0.77), while there were nonsignificant group differences in the other nine emotions (*p* > 0.05 in all the *t*-tests).

The results of the pilot study supported that the adapted AEMT is an efficient method of arousing the target emotion (i.e., anger), and more specifically, anger that was caused by another person. After writing the AEMT participants in the anger condition felt significantly higher levels of anger, while experiencing comparable levels of the other nine emotions, relative to those in the control condition. Therefore, the advantage of this method is that it can avoid arousing the non-target emotions, i.e., side effects on non-target emotions (Mills and D'Mello, 2014).

## Study 1

Study 1 aimed at investigating the effect of anger on trust behavior by applying the adapted and previously tested AEMT to arouse anger. More precisely, we explored the proposed moderated mediation model in this study, which the effect of anger on trust was mediated by perceived social distance, and this mediation was moderated by gender (Hypothesis 1).

### Method

#### Participants and Data Collection

A total of 210 German university students (51.0% female) voluntarily participated in this study. The participants were recruited using the online recruiting system ORSEE (Greiner, 2015). They were compensated by a fixed participation fee (3 €) plus variable payments according to their individual decisions in the trust game (theoretical range: 0–12 €), which on average resulted in a pay of 7.56 € for a 30-min experiment. The assignment to the treatment conditions was random with *N* = 98 participants in the anger condition (51.0% female) and *N* = 112 in the control condition (50.9% female), with no significant difference in the age of participants in the two conditions (*t* = 0.61, *p* = 0.54, Anger: *M* = 21.31, *SD* = 2.33, Control: *M* = 21.52, *SD* = 2.65).

#### Experimental Design and Procedure

##### Experimental Design

This study used a 2 (Anger vs. Control) × 2 (Men vs. Women) factorial design. Participants completed a different version of the AEMT based on the condition they were in, the anger or control condition. Participants were then paired with a stranger to play the trust game. Finally, anger, perceived social distance, as well as social-demographic variables (e.g., general trust belief in other people, sex, age, subject of study, monthly disposable money, pre-experiences with computer-games) were measured after playing the trust game. The experiment was programmed using *z*-Tree (Fischbacher, 2007).

##### AEMT

Study 1 used the adapted version of the AEMT (Strack et al., 1985; Mills and D'Mello, 2014), which was tested in the pilot study.

##### The Trust Game

**Figure 2:**
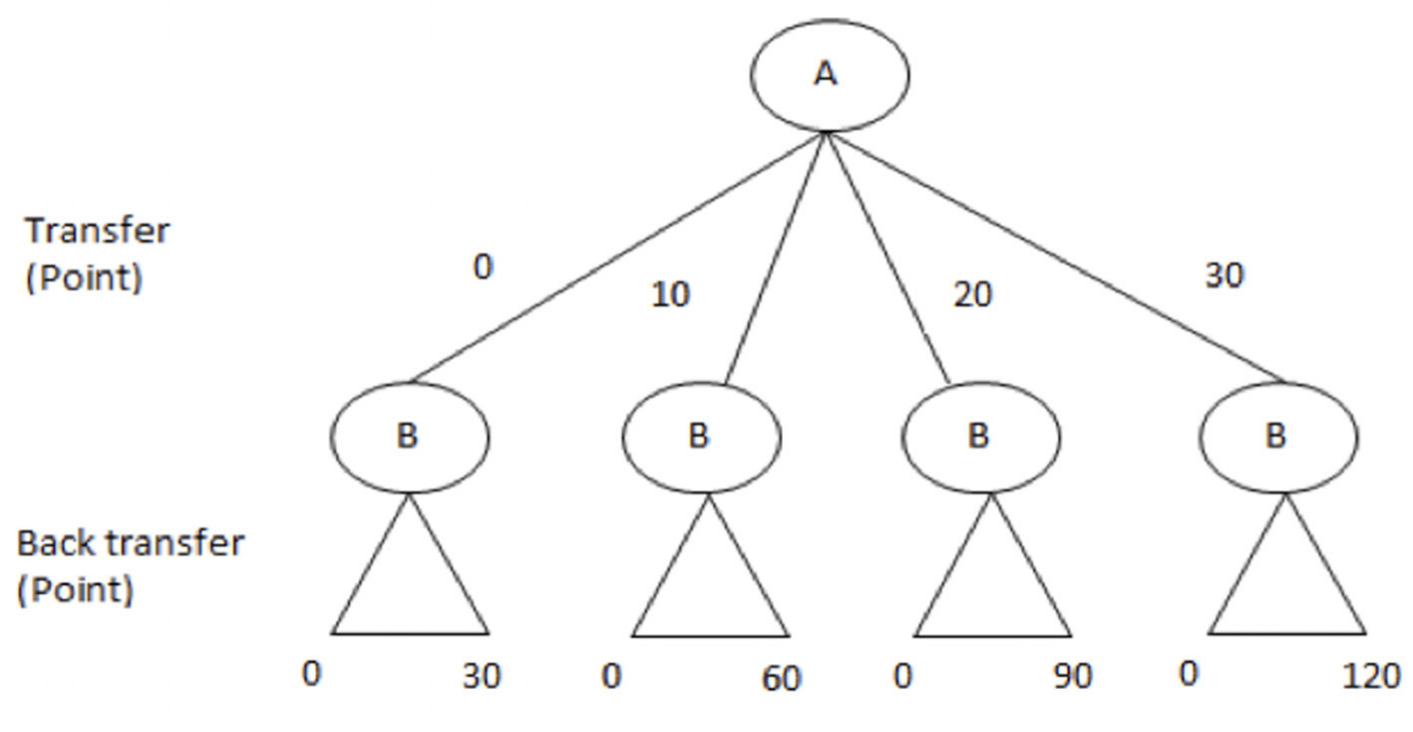
The trust game. There are two players (*A* and *B*) in this game. Both *A* and *B* will receive an initial endowment of 30 points from the experimenter. A can choose whether to send 0, 10, 20, or 30 points to *B*. Whatever amount *A* decides to send to *B* will be tripled by the experimenter before it is passed on to *B*. *B* then has the option of returning any amount between zero and his/her total amount of available points to *A*. For example, if *A* has sent 10 point, *B* possesses 60 points (30 points own endowment + 30 points tripled transfer) and can, therefore choose any back transfer from 0 to 60 points. The experimenter does not triple the back transfer.

An investment game (Berg et al., 1995) was applied to assess participants' trust. In this game, there are two players (*A* and *B*), both are anonymous and randomly paired to each other. They are informed that they will interact with each other only once. Both *A* and *B* will receive an initial endowment of 30 points (1 point = 0.10 €) from the experimenter. *A* then has the opportunity to give a portion of his/her points to *B*. *A* can choose whether to send 0, 10, 20, or 30 points to *B*. Whatever amount *A* decides to send to *B* will be tripled by the experimenter before it is passed on to *B*. *B* then has the option of returning any amount between zero and his/her total amount of available points to *A*. For example, if *A* sends 10 points, they are tripled to 30 points before they are passed on to *B*. Then *B* possesses 60 points (30 points own endowment + 30 tripled points) and can choose any back transfer from 0 to 60 points (see Figure 2). The experimenter does not triple the back transfer. All participants start the game as player *A*. Only after they finish the decision of *A*, they are informed to play the role of *B* as well (Burks et al., 2003). Player *A*'s decision in the trust game represented the trust behavior. The final payoff of player *A* corresponds to the initial endowment minus the transfer to *B*, plus the back transfer from *B*. The final payoff of player *B* is given by his initial endowment plus the tripled transfer of *A*, minus the back transfer to *A*. At the end of the experiment, we randomly choose one of participants in each session to roll a die to decide which role (as player *A* or *B*) of them would be paid in this game. The earned points are exchanged into real money according to a publicly announced exchange rate.

#### Variables and Study Measures

##### Anger

Applying the subscales of the Differential Emotion Scale (Izard et al., 1974; German version: Merten and Krause, 1993), anger was assessed by self-report measures after the trust game as a manipulation check. The subscale consists three adjective items (e.g., “enraged”, “angry” “mad”). Participants' anger was assessed by their ratings on a five-point intensity rating scale ranging from 0 *not at all* to 4 *very strong*. The internal consistency of anger was high, α = 0.94. Furthermore, anger attribution was adapt as a manipulation check to examine whether anger was caused by the self or others or something beyond anyone's control (Smith and Ellsworth, 1985).

##### Social Distance

We adopted the Inclusion of Other in the Self (IOS) Scale (Aron et al., 1992) to measure the social distance that participants perceived toward their game partner in the trust game, which is a widely used as a measure of relationship closeness (e.g., Tan et al., 2015; Helgeson and Van Vleet, 2019; Pietras and Briken, 2020). Participants rated the social distance toward their game partner on a set of seven Venn-like diagrams; it ranged from 1 (small social distance) to 7 (large social distance). The social distance that participant perceived as player *A* was measured.

### Results

#### Anger

Following the experimental manipulation, participants in the anger condition showed significantly higher levels of anger than participants in the control condition (β = 2.14, *t* = 12.05, *p* = 0.000, *d* = 2.22; anger: *M* = 2.40, *SD* = 1.17; control: *M* = 0.35, *SD* = 0.63). Furthermore, the main effect of gender (β = −0.15, *t* = −0.86, *p* = 0.394), as well as the interaction of gender and experimental manipulation on anger were not significant (β = −0.18, *t* = −0.72, *p* = 0.473). Furthermore, anger attribution as a manipulation check indicated that the decision maker and the counterpart are not the source of anger. Therefore, results showed the experimental manipulation of anger was successful and induced similar intensities of anger for males and females.

#### Trust

**Figure 3:**
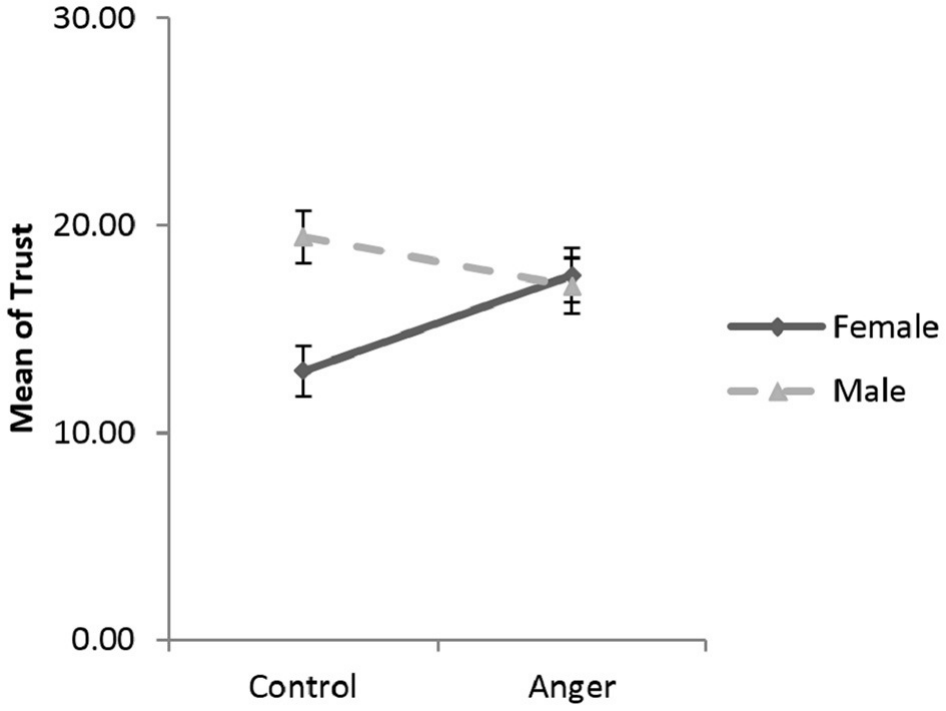
Estimated marginal means of trust (in point) in the anger and control conditions in Study 1. Error bar is the standard error.

In the present study, player *A*'s decision in the trust game represented the trust behavior. We used a linear regression to assess the effects of the experimental condition (Anger, Control) and gender on trust (Hayes, 2013). The experimental condition (anger = 1, control = 0) and gender (male = 1, female = 0) were dummy coded. The regression was significant, *R*^2^ = 0.07, *F* (3, 206) = 4.89, *p* = 0.003, $\eta_{\text{p}}^{2}$ = 0.03, 1–β = 0.78. Both the regression coefficients of the experimental condition (β = 4.62, *t* = 2.58, *p* = 0.011) and the one for gender (β = 6.47, *t* = 3.71, *p* = 0.000) were significant, and a significant interaction between the experimental condition and gender was observed (β = −6.99, *t* = −2.73, *p* = 0.007; see Figure 3).

The simple slopes method (Aiken et al., 1991) was used to investigate this interaction in detail. The results showed that women were more trusting in the anger condition than in the control condition (β = 4.62, *t* = 2.58, *p* = 0.011, *d* = 0.55). Female participants in the anger condition were 20.7% more likely to send the maximum option (30 point) and 10.0% less likely to send the minimum option (0 point), as compared to females in the control condition (Kolmogorov-Smirnov test; *Z* = 1.07, *p* = 0.035). For males, the mean of trust (β = −2.37, *t* = −1.30, *p* = 0.195, *d* = −0.24) and the distribution of trust (Kolmogorov-Smirnov test; *Z* = 0.85, *p* = 0.174) were not significantly different across the experimental conditions. Men were more trusting than women in the control condition were (β = 6.47, *t* = 3.71, *p* = 0.000, *d* = 0.73), while there was no gender difference in the anger condition (β = −0.52, *t* = −0.28, *p* = 0.782, *d* = −0.05).

#### Moderated Mediating Effect of Anger on Trust Through Social Distance

In order to understand why women but not men were more trusting in the anger condition than in the control condition, we examined whether social distance mediated the relationship between anger and trust, and whether the magnitude of this indirect effect was different for men and women. Thus, a moderated mediation model was used (Preacher et al., 2007). The proposed moderated mediation model was modeled by three paths leading from the experimental condition (independent variable), toward perceived social distance (mediator) and trust (dependent variable), as well as from perceived social distance toward trust, and gender (moderator) moderated the path from experimental condition to social distance (see Figure 1).

The moderated mediation model was significant, *R*^2^ = 0.21, *F* (2, 207) = 28.14, *p* = 0.000, bootstrap samples = 10,000 (Table 1). The results revealed a significant interaction between experimental condition and gender on perceived social distance (β = 0.77, *t* = 2.85, *p* = 0.005), which means that the effect of experimental condition on participants' perceived social distance was moderated by gender. Women in the anger condition perceived smaller social distance between themselves and their game partners than women in the control condition (β = −0.37, *t* = −1.96, *p* = 0.052, *d* = 0.44; anger: *M* = 5.02, *SD* = 1.57; control: *M* = 5.64, *SD* = 1.27); while men in the anger condition perceived larger social distance between themselves and their game partners than men in the control condition (β = 0.40, *t* = 2.07, *p* = 0.040, *d* = −0.36; anger: *M* = 5.19, *SD* = 1.83; control: *M* = 4.51, *SD* = 1.91). Furthermore, the results shown in Table 2 revealed that the indirect effects of experimental condition on trust, via perceived social distance, was significantly positive for women (β = 1.61, 95% CI [0.201, 3.177]) but not for men (β = −1.74, 95% CI [−3.785, 0.139]). The direct effect of experimental condition on trust was not significant (β = 1.22, 95% CI [−1.088, 3.525]).

**Table 1:** Model coefficients for conditional indirect effect of the experimental condition on trust through perceived social distance in Study 1.

	**SD_A**			**Trust**		
**Antecedent**	***Coeff*.**	***SE***	***p***	***Coeff*.**	***SE***	***p***
*Constant*	0.32	0.13	0.013	16.14	0.80	0.000
Dummy_AC	−0.37	0.19	0.052	1.22	1.17	0.299
SD_A	–	–	–	−4.34	0.58	0.000
Gender	−0.67	0.19	0.000	–	–	–
Dummy_AC × Gender	0.77	0.27	0.005	–	–	–
	*R^2^* = 0.061			*R^2^* = 0.214		
	*F* (3, 206) = 4.49,			*F* (2, 207) = 28.14,		
	*p* = 0.004			*p* = 0.000		

*Dummy_AC: Anger condition = 1, Control condition = 0; Gender: Male = 1, Female = 0; SD_A: Social distance be perceived as A (player A in the trust game). SD_A was standardized. Bootstrap samples = 10,000*.

**Table 2:** Direct and conditional indirect effects of the experimental condition on trust in Study 1.

	**Dummy_AC on Trust**			
***Effects***	***Gender***	***Coeff*.**	***SE***	**95% Bias-corrected Bootstrap CI**
Direct	–	1.22	1.17	−1.088 to 3.525
Indirect	0	1.61	0.76	0.201 to 3.177
Indirect	1	−1.74	0.99	−3.785 to 0.139

*Dummy_AC: Anger condition = 1, Control condition = 0; Gender: Male = 1, Female = 0*.

### Discussion

This study provides support for Hypothesis 1, which states that the effect of anger on trust will be positively mediated by participants' perceived social distance to their partners, and this mediation will be moderated by gender. In line with our hypothesis, the results showed that women were driven by anger to perceive smaller social distance, and consequently sent more money to their interaction companion as compared to controls. On the other hand, men perceived larger social distance in the anger condition than in the control condition, while they sent a similar amount of money in both conditions. One possible explanation could be that trust of women, but not men, would depend on their perceived social distance. Therefore, in Study 2 we experimentally manipulated social distance in order to further examine the mediational role of perceived social distance in the relationship between anger and trust (Spencer et al., 2005).

## Study 2

In Study 2, we aimed to extend the results of Study 1 by critically testing the causal role of the mediator, that is, to examine the effects of perceived social distance on trust (Spencer et al., 2005; MacKinnon et al., 2007). We experimentally manipulate social distance, by either allowing (i.e., low social distance) or not allowing (i.e., control) participants to engage in some interaction in the form of online chatting prior to engaging in the trust game (Buchan et al., 2006). In line with the results of Study 1, we predict that women, but not men, will send more money to their game partner in the low social distance condition than in the control condition.

### Method

#### Participants and Data Collection

A total of 106 German university students (46.2% female) voluntarily participated in this study. The participants were recruited using the online recruiting system ORSEE (Greiner, 2015). They were compensated by a fixed participation fee (3 €) plus variable payments according to their individual decisions in the trust game (theoretical range: 0 – 12 €), which on average resulted in a pay of 7.4 € for a 30-minute experiment. The assignment to the treatment conditions was random with *N* = 52 participants in the low social distance condition (48.1% female) and *N* = 54 in the control condition (44.4% female), with no significant differences in the age of participants in the two conditions (*t* = 0.52, *p* = 0.60, Low social distance condition: *M* = 21.10, *SD* = 3.46, Control: *M* = 21.41, *SD* = 2.62).

#### Experimental Design and Procedure

##### Experimental Design

This study used a 2 (Low social distance vs. Control) × 2 (Men vs. Women) factorial design. Participants were randomly assigned to either a low social distance or a control condition, with balanced gender. First, the social distance of the trustee was manipulated. Then, participants were paired and instructed to play the same trust game as in the Study 1. The perceived social distance of trustee and socio-demographic variables (e.g., general trust belief in other people, gender, age, program of study, monthly disposable income, and previous experience with computer-games) were measured after playing the trust game. We measured the perceived social distance by the same IOS Scale as in Study 1 (Aron et al., 1992). The experiment was programmed using z-Tree (Fischbacher, 2007).

##### Chatting Task

The social distance of the trustee was manipulated via an online chatting task. In the low social distance condition, four anonymous participants in each session were in one chatting group. They could chat about either one of the three suggested topics (your favorite sports, your favorite holiday or a memorable birthday celebration; Buchan et al., 2006; Fiedler et al., 2011) or any other topics, while staying anonymous. They had five minutes to talk with their group members in the on-line chatting program. In the control condition, participants had no communication with one another, instead they were asked to “*Describe in detail the* ***mundane events*** *of the previous day”* (Bodenhausen et al., 2000). It is important to note that the communications in the low social distance condition could not have been related to the trust game, as participants did not know they were going to play a trust game later (Buchan et al., 2006). In the low social distance condition, participants were then informed that they would play the trust game with someone random from his/her chatting group, while in the control condition, the game partner was someone random from the same session of the experiment.

### Results

**Figure 4:**
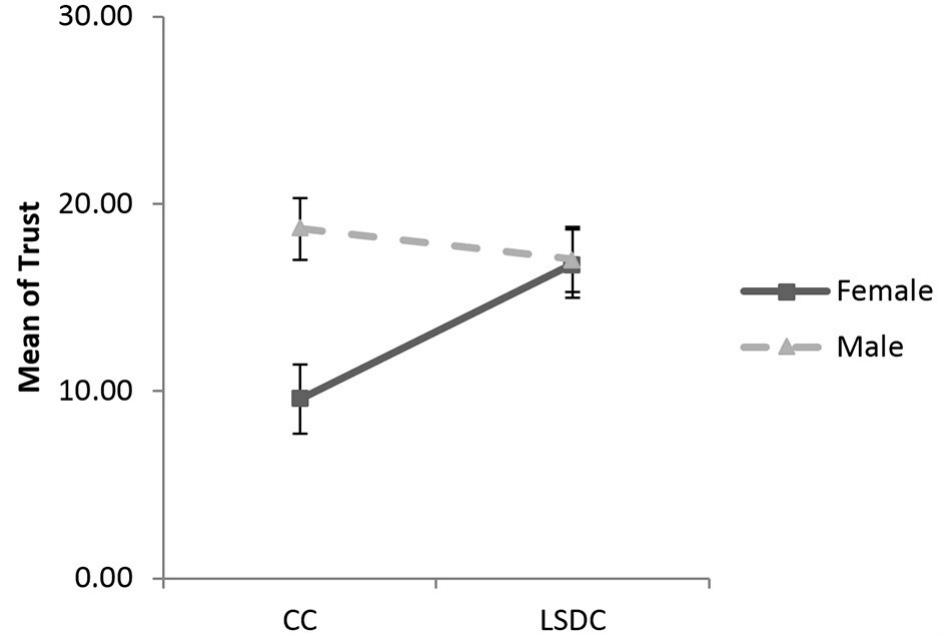
Estimated marginal means of trust (in point) in the control condition (CC) and low social distance condition (LSDC) in Study 2. Error bar is the standard error.

We used a linear regression to assess the effects of the social distance condition and gender on trust (Hayes, 2013). The social distance condition (low social distance condition = 1; control condition = 0) and gender (male = 1; female = 0) were dummy coded, and age was added as covariant. The regression was significant, *R*^2^ = 0.135, *F* (4, 101) = 3.95, *p* = 0.005, $\eta_{\text{p}}^{2}$ = 0.05, 1–β = 0.70. The regression coefficient of the social distance condition (β = 7.43, *t* = 2.87, *p* = 0.005) and the one for gender (β = 9.11, *t* = 3.67, *p* = 0.000) were significant, and the interaction between the social distance condition and gender was also significant (β = −9.12, *t* = −2.57, *p* = 0.012; see Figure 4).

The simple slopes method (Aiken et al., 1991) was used to investigate this interaction in detail. The results revealed that women were more trusting in the low social distance condition than in the control condition (β = 7.43, *t* = 2.87, *p* = 0.005, *d* = 0.90). Female participants in the low social distance condition were 20% more likely to send the maximum option (30 point) and 8.8% less likely to send the minimum option (0 point), as compared to females in the control condition (Kolmogorov-Smirnov test; *Z* = 1.52, *p* = 0.003). For males, the mean of sent points (β = −1.68, *t* = −0.70, *p* = 0.485, *d* = −0.17), as well as the distribution of sent points (Kolmogorov-Smirnov test; *Z* = 0.42, *p* = 0.680) were not significantly different across the social distance conditions.

### Discussion

This study critically tested the causal role of the mediator, i.e., perceived social distance, on trust (Spencer et al., 2005; MacKinnon et al., 2007). Results of Study 2 support Hypothesis 2. For female participants, a reduced perceived social distance resulted in them exhibiting more trusting behaviors. This pattern of results was not found in male participants, which confirmed results from Study 1 that men's trust might not dependent on perceived social distance.

## General Discussion

### Findings and Contributions

The willingness of individuals to trust others is fundamental to the economic and social life of all societies (e.g., Greenspan, 1999; Algan and Cahuc, 2013). Trust has been viewed as a deliberate act based on thorough cognitive calculations, by weighting the costs and benefits of certain actions (e.g., Williamson, 1993; Fetchenhauer and Dunning, 2009). While many trust-related behaviors are made in emotion-rich environments (Dunning et al., 2012; Engelmann and Fehr, 2013), the role of anger in the decision-making process of whether a person is trustworthy is still mostly unclear. Is it possible that anger overrides rationality and induces individuals to trust others “blindly”? To answer this question, the present study investigated how anger impacts subsequent trust behavior, as well as the roles that gender and social distance play in this relationship. By using a classic directed-writing task to manipulate anger, we confirmed the predicted moderated mediation model. Namely, the effect of anger on trust was positively mediated by perceived social distance, and this mediation was moderated by gender. Furthermore, we conducted a second study to test the causal role of the mediator, and provided further evidence for the effect of perceived social distance on trust. Therefore, the present results contribute to a deeper understanding of the literature on trust and emotions, and more specifically, the role of anger in trust-related decisions.

The subjective experience of anger typically increases individuals' propensity to seek out pleasant and rewarding stimuli that are unrelated to the source of the anger (Ford et al., 2010; Angus et al., 2015). The positive effect of anger on women's trust is consistent with the assertion that anger increases individual's confidence and encourages them to actively approach a situation (e.g., Lerner and Tiedens, 2006; Carver and Harmon-Jones, 2009). The approach motivation that was associated with anger in this study was reflected in female participants perceiving smaller social distance to their interaction companions, and it was that perceived social distance that drove participants to send money to their interaction partners. We further tested the causal effect of social distance on trust, and results revealed that only female participants were influenced by their perceived social distance when making their trust decision.

Moreover, our results are in line with previous evidence showing that women's trust is more context-sensitive than men's (see review, Eckel and Wilson, 2004; Croson and Gneezy, 2009). Findings from the present study may also contribute to the understanding of results from previous studies (e.g., Chaudhuri and Gangadharan, 2007; Buchan et al., 2008; Garbarino and Slonim, 2009; Charness and Gneezy, 2012) that found women to be less trusting and more financially risk averse than men while others didn't find these gender differences (e.g., Cox and Deck, 2006; Schwieren and Sutter, 2008). Our results indicate that anger may transform the previously uncovered risk aversion into reward-seeking in the process of trust, thus elevating women's measured trust to the same level as men's (Angus et al., 2015). Although similar trust games were used in other studies, the experimental settings and/or conditions were different from the ones discussed here, therefore, gender differences may not be observed in the trust process. We believe that the existence of gender differences in the emotional experiences and/or perceived social distance in the process of actual trust behavior can cause the inconsistent gender differences found in the trust literature. Future research should consider these gender differences when investigating trust behavior, particularly when focusing on women's trust.

The current findings did not support the findings by Dunn and Schweitzer (2005) which demonstrate that control appraisals of anger determined whether or not anger would decrease trust behavior. There were two main differences between our experimental setups and the previous findings that might account for these inconsistencies. First, Dunn and Schweitzer's (2005) argued that anger theoretically was an emotion with “other-person control” appraisal, but the authors neither specified whether the anger was caused by another person in their manipulation, nor checked whether participants in their experiments held an “other-person control” appraisal or “self-/situational-control” appraisal. We manipulated anger to be specifically caused by another person, and found positive effects of anger on trust in women, which contradicted Dunn and Schweitzer's (2005) argument. Secondly, Dunn and Schweitzer (2005) measured trust using a survey instrument which was similar to the trust beliefs measure, but did not measure participants' actual trust behavior. In the present studies, we operationalized trust via the trust game, which is a classic game in capturing the actual trust behavior (Johnson and Mislin, 2011). As noted previously, survey-measures of trust are not always congruent with real trust as it is demonstrated by the actions taken during the trust game (e.g., Eckel and Wilson, 2004; Fetchenhauer and Dunning, 2009).

### Limitation and Future Research

The present studies provide support for the influence of anger on trust behavior, although some findings are near the *p* = 0.05 level, we think that they are rather meaningful in nature. Two limitations should be taken into account. First, the present studies focus on anger but not on other emotions, providing evidence as to the mechanism by which anger influences trust via the role of gender and perceived social distance. Future research should further investigate whether this mechanism can be generalized to other emotions. For instance, future research should investigate whether discrete emotions, like anger (high certainty and associated approach motivation) and fear (low certainty and associated avoidance motivation), differentially affect trust behavior, and whether these effects are mediated by perceived social distance. Lerner and Keltner (2000, 2001), Lerner et al. (2004) found that angry people were risk-seeking while fearful people were risk-averse in their risk-taking behavior. However, trust actions are above and beyond people's risk preference (e.g., Bohnet and Zeckhauser, 2004; Bohnet et al., 2008), as it involves the uncertainty of another person's actions. Therefore, here we suggest to take into account how these discrete emotions shape people's perceived social distance to others in the actual trust process. Furthermore, the current study measured the anger based on participants' self-report, which might cause side effect of social desirability or courtesy bias in contributing to the observed difference between anger and control conditions. Future research could apply both self-report and behavioral paradigms (e.g., Beisswingert et al., 2015) to measure anger, to confidently rule out the potential desirability or courtesy bias in influencing the effects of anger.

Second, the social distance of the trustee in Study 2 was manipulated through the creation of artificial groups for online chatting, but not based on naturally-occurring groups (Buchan et al., 2006). These artificial groups may restrict variances in experienced social distance, as compared with the function of natural groups (e.g., families vs. strangers) in manipulating of social distance. Therefore, it is still not clear whether men's trust is contingent on the perceived social distance that was aroused by artificial groups. Future research could investigate how perceived social distance, experienced with naturally-occurring social groups or in more experimentally-manipulated settings, influence trust.

## Conclusion

The present study shows that anger drove women, but not men, to send more money to their counterparts in a trust game, and this effect was mediated by perceived social distance. Furthermore, this study critically tested the causal role of perceived social distance on trust and found that women, but not men, trusted their game partners more in the low social distance condition than in the control condition. These findings provide some initial insight into the mechanism behind how anger influence human trust behavior. Although anger has been classified with respect to its effects as a “negative” emotion (for reviews, Ben-Ze'ev, 2001; Berkowitz and Harmon-Jones, 2004), our findings may cast a more positive light on it. Anger does not follow many of the typical patterns associated with negative emotions; rather than triggering less trust, it triggered more trust for women when it was expressed in a non-destructive way. This finding is in line with previous research that shows the rather positive aspects of anger (Lerner and Keltner, 2000, 2001; Loewenstein and Lerner, 2003; Carver and Harmon-Jones, 2009; Beisswingert et al., 2015). However, although there is a number of seemingly advantageous effects of anger, it is an emotion which is subjectively experienced as an unpleasant feeling (e.g., Ben-Ze'ev, 2001), and which people try to avoid. This makes anger a highly interesting emotion, and one that is worthwhile further investigation.

## Data Availability Statement

The datasets presented in this study can be found in online repositories. The names of the repository/repositories and accession number(s) can be found here: osf.io/urs5j.

## Ethics Statement

The studies involving human participants were reviewed and approved by University of Konstanz. The patients/participants provided their written informed consent to participate in this study.

## Author Contributions

Conceived and designed the experiments: KZ, TG, and FC. Performed the experiment and Literature research: KZ. Analyzed the data: KZ and TG. Contributed materials/analysis tools: KZ and FC. Wrote the paper: KZ, TG, and AS. All authors contributed to the article and approved the submitted version.

## Conflict of Interest

The authors declare that the research was conducted in the absence of any commercial or financial relationships that could be construed as a potential conflict of interest.
